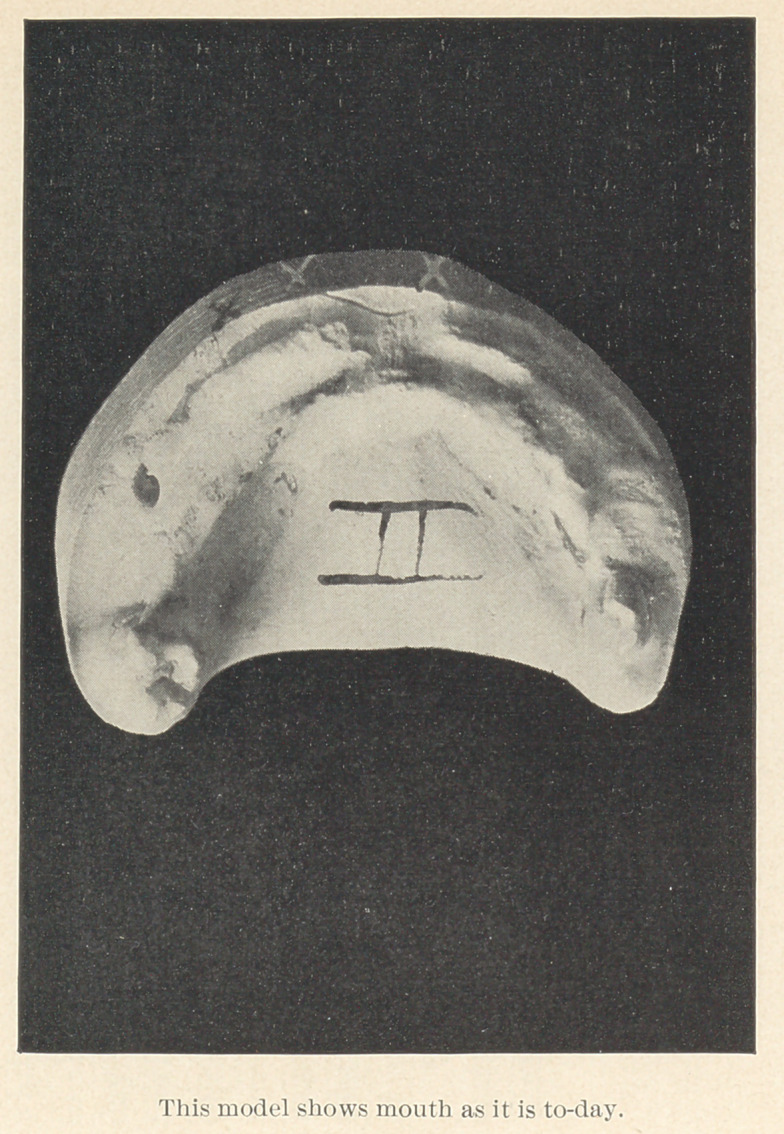# Case of Arsenical Necrosis

**Published:** 1902-11

**Authors:** James Edward Power

**Affiliations:** Providence, R. I.


### CASE OF ARSENICAL NECROSIS.

BY JAMES EDWARD POWER, D.M.D., PROVIDENCE, R. I.[1]

I REPORT one of the many cases which have come under my observation during the past few years at the hospitals at which I am connected, wherein it is reasonable to suppose that had the practitioners who were first called to see this, and similar cases, conformed to the laws of the proverb, "Success seeks to crown him

[1] Assistant Dental Surgeon to Rhode Island and St. Joseph's Hospitals, Providence, R. I.

who is firm in his conviction, but invites reason to opinions he holds or expresses, and who realizes that the field of knowledge is far greater than his own personal store," much suffering would have been obviated.

This applies to every man, and especially to men who are engaged in professional work, because no man, no matter how proficient, can possess a monopoly on all the important branches, whether it be diagnosis or treatment.

With all due respect to the practitioner who first treated this case, is it unreasonable to feel that this poor man had been done an injustice, knowing the destruction this disease will cause in twenty-four or forty-eight hours, and considering that it was allowed to progress for thirty-five days without operation, and nothing to check its progress but applications of hot flaxseed poultices and salt-bags?

I obtained the following history from the patient:

Mr. H., aged thirty-six years; born in New York; occupation, dresser tender. Previous to the time of his illness he was always healthy and robust; mouth never gave him any trouble, as all teeth were present except two molars on each side of the inferior maxilla.

On the evening of May 8, 1902, patient, on returning home after the day's work, complained of feeling sick,—headache and nausea. His condition remained about the same until Sunday, May 11, 1902, when he noticed a dull pain in the region of the symphysis and extending all along the body of the bone. He thought it was neuralgia, but the pain developed so during the day that he resorted to one grain of morphine that night to relieve the pain, which by this time was almost unbearable, and caused him to lie awake all night.

During the following night he took five quarter-grain tablets, but pain continued, and he was very delirious during this and the following night.

On May 14, 1902, a physician was called, who found the patient's temperature 105° F. He diagnosed the trouble neuralgia, and advised application of hot salt- and water-bags to the chin. This treatment was continued for eight days, during which time the skin covering the chin was destroyed by the heat, the inflammation on the inside of his mouth was increased, and the physician incised the gum freely, not getting any or much pus. The patient seemed to get temporary relief while the heat was applied to his face, but on removal of same, pain was much worse than before.

May 15, the physician changed his diagnosis and said it was an ulcerated tooth, and extracted inferior central incisor tooth.

May 16, extracted lateral incisor tooth.

May 20, he advised mouth-wash, and on May 22 patient requested physician to discontinue his calls, as he seemed to be no better than he was on May 14.

On May 23 he visited an emergency hospital. Temperature, 102.7° F. The attending physician said he did not know what was the trouble, but advised application of hot flaxseed poultice. After five days, abscess appeared and was lanced. Patient continued to syringe the mouth until about the first week in June, when he was referred to a dentist for advice. The dentist refused to extract the tooth, as he said there was no cavity.

About a week later, he visited another dentist, who extracted this tooth, and three days later extracted three more.

The patient visited the Emergency Hospital until June 11, 1902. On this day he was told to visit St. Joseph's Hospital for advice by the attending physician.

On June 12 he was among the patients at my clinic at St. Joseph's Hospital, and examination presented a well-developed case of necrosis. Inside, the mouth was highly inflamed, and on the chin in the region of the symphysis was a very tender spot, which gave the patient a great amount of pain on being touched very lightly with the finger. His condition was poor, he having lost twenty-two pounds since May 14. I advised immediate operation, and on Saturday morning, June 14, I proceeded to operate under ether, first extracting the remaining teeth,—two bicuspids on the right side and one bicuspid on the left, leaving the two last molars present.

Although the area that seemed to be affected was confined to the body of the bone previously occupied by the six anterior teeth, I made an incision along the body of the bone (inside) from one wisdom-tooth to the other, and by the use of spoon-shaped curettes scraped all dead bone away. I then cut all sharp or jagged edges away with bone-cutters. I prescribed mouth-wash, and did not see patient again until half-past ten in the evening; found him quite comfortable. Temperature normal the following day. I visited him every day until July 20, when I discharged him, cured.

This model was taken shortly after operation, and carved to show area of bone which was affected. Crosses (×) show places where taken with prote. from the superior to the inferior surface, and measurement was one inch deep.

This model shows mouth as it is to-day.

An interesting and peculiar thing connected with this case was that although the tissue posterior to the bicuspid teeth seemed normal, the bone was affected very extensively.

A good rule to use in operating on necrotic bone is one I learned during my second year at college, and which I think may be applied to all cases where a cure is desired by operation,—namely, scrape until you are sure you have scraped enough, and then scrape some more.

When I first saw the patient I was somewhat baffled as to the cause and the special kind of necrosis with which he was afflicted. On answering my questions, he said he had never visited a dentist, had received no recent injury to the part, had never held matches in his mouth, and had never had syphilis. Family history good; no tuberculosis; had his chest examined for same, and the interne who made the examination found his lungs in excellent condition. This information removed from the list of probable causes necrosis from broken bone, necrosis from arsenic used by dentists, syphilitic necrosis, phosphorus necrosis, and tubercular necrosis.

Finally, I asked him what he used to cleanse his teeth, thinking that he may have become infected from the use of cheap dentifrices sold by "fakirs," which, when applied, "change black teeth to white instantaneously," but he replied that he never used anything to clean his teeth except pieces of yarn. He used this about every day to remove particles of food which became lodged between the teeth. Closer investigation showed that he used yarn of all shades, especially green and red. I then concluded that he had become infected from the arsenic used in dyeing the yarn, and requested him to bring some of the yarn to me. I examined same and found that it did contain arsenic, thus removing all doubt as to the kind of necrosis with which my patient was affected.

The patient is now entirely cured, and is wearing a plate to restore contour of face and to give him the advantage of artificial teeth also.

## Figures and Tables

**Figure f1:**
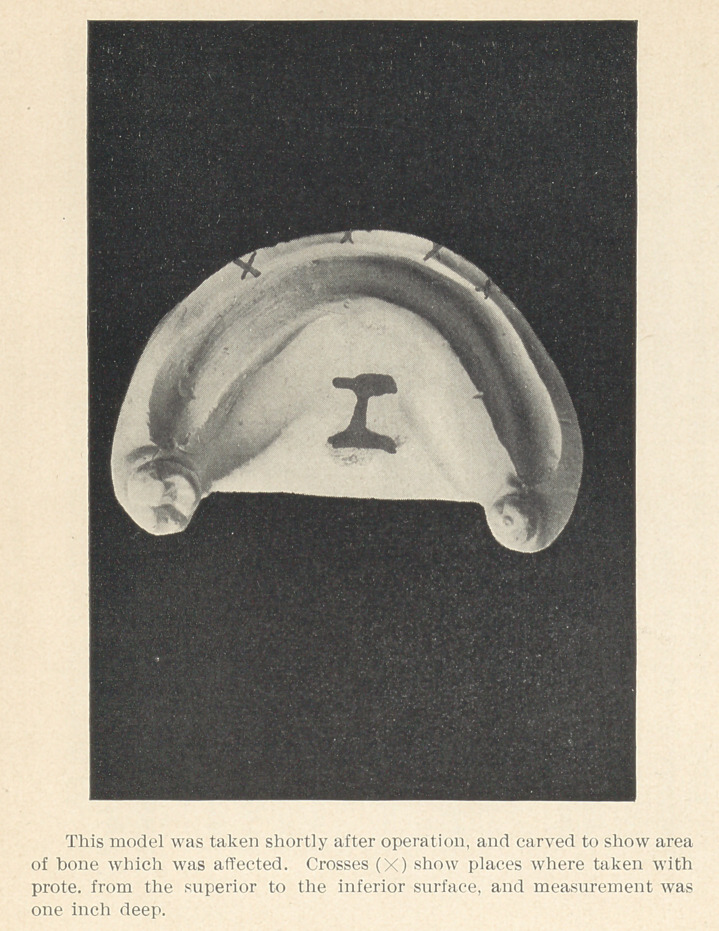


**Figure f2:**